# Utilization of computer vision and multispectral imaging techniques for classification of cowpea (*Vigna unguiculata*) seeds

**DOI:** 10.1186/s13007-019-0411-2

**Published:** 2019-03-12

**Authors:** Gamal ElMasry, Nasser Mandour, Marie-Hélène Wagner, Didier Demilly, Jerome Verdier, Etienne Belin, David Rousseau

**Affiliations:** 1 Agricultural Engineering Department, Faculty of Agriculture, Suez Canal University, P.O Box 41522, Ismailia, Egypt; 2 GEVES, Station Nationale d’Essais de Semences (SNES), 49071 Beaucouzé, Angers, France; 3 Laboratoire Angevin de Recherche en Ingénierie des Systèmes (LARIS), Université d’Angers, Angers, France; 4 INRA, UMR1345 Institut de Recherche en Horticulture et Semences, 49071 Beaucouzé, Angers, France

**Keywords:** Cowpea seeds, Black-eyed seeds, Plant development, Germination, Phenotyping, Chemometric, Multispectral imaging

## Abstract

**Background:**

The traditional methods for evaluating seeds are usually performed through destructive sampling followed by physical, physiological, biochemical and molecular determinations. Whilst proven to be effective, these approaches can be criticized as being destructive, time consuming, labor intensive and requiring experienced seed analysts. Thus, the objective of this study was to investigate the potential of computer vision and multispectral imaging systems supported with multivariate analysis for high-throughput classification of cowpea (*Vigna unguiculata*) seeds. An automated computer-vision germination system was utilized for uninterrupted monitoring of seeds during imbibition and germination to identify different categories of all individual seeds. By using spectral signatures of single cowpea seeds extracted from multispectral images, different multivariate analysis models based on linear discriminant analysis (LDA) were developed for classifying the seeds into different categories according to ageing, viability, seedling condition and speed of germination.

**Results:**

The results revealed that the LDA models had good accuracy in distinguishing ‘Aged’ and ‘Non-aged’ seeds with an overall correct classification (OCC) of 97.51, 96.76 and 97%, ‘Germinated’ and ‘Non-germinated’ seeds with OCC of 81.80, 79.05 and 81.0%, ‘Early germinated’, ‘Medium germinated’ and ‘Dead’ seeds with OCC of 77.21, 74.93 and 68.00% and among seeds that give ‘Normal’ and ‘Abnormal’ seedlings with OCC of 68.08, 64.34 and 62.00% in training, cross-validation and independent validation data sets, respectively. Image processing routines were also developed to exploit the full power of the multispectral imaging system in visualizing the difference among seed categories by applying the discriminant model in a pixel-wise manner.

**Conclusion:**

The results demonstrated the capability of the multispectral imaging system in the ultraviolet, visible and shortwave near infrared range to provide the required information necessary for the discrimination of individual cowpea seeds to different classes. Considering the short time of image acquisition and limited sample preparation, this stat-of-the art multispectral imaging method and chemometric analysis in classifying seeds could be a valuable tool for on-line classification protocols in cost-effective real-time sorting and grading processes as it provides not only morphological and physical features but also chemical information for the seeds being examined. Implementing image processing algorithms specific for seed quality assessment along with the declining cost and increasing power of computer hardware is very efficient to make the development of such computer-integrated systems more attractive in automatic inspection of seed quality.

## Introduction

Cowpea (*Vigna unguiculata*) known also as black-eyed pea and originated in Africa is a very strategic crop in the world having a total harvested area of 12.3 million hectares with an annual dry seed production of 7.0 Mt and an average yield of 5676 kg ha^−1^ [1]. Cowpea seed is a nutritious component in the human diet and livestock feed as it is rich in the amino acids, lysine and tryptophan compared to cereal grains [2]. Nowadays, there is an increasing concern from all governmental organizations to increase productivities of arable lands by providing seeds of high quality. Seed quality is an important issue for all stakeholders involved in crop production including breeders, producers, traders, variety registration agencies, farmers and distributers. The growing markets in seed business at national and international levels have stimulated great interest in selecting the best descriptive characterization of seed quality. The concept of seed quality is composed of several attributes, including varietal and genetic purity, viability, health, germination capacity, vigor and uniformity [3]. Besides morphological and phenotypic features, some other important parameters such as biochemical, genotypic and molecular markers are very important also to fulfill. In fact, without good seeds the investment on fertilizers, water, pesticides, and other inputs will not be worth.

The growing demand for reliable details about quality and conditions of seed properties has induced suppliers to improve the information provided about the seed quality traits throughout intensive seed testing scenarios. Some of this information is very easy to achieve; meanwhile, other information related to viability, damage, vigor and health is very difficult to obtain directly due to time and technological constraints. Seed testing is generally based on the detection of various physical, physiological, biochemical and, more recently, biomolecular markers which correlate well with seed constituents [4]. The ordinary practices of seed quality evaluation are usually made through germination test and can be complemented by vigor tests and seedling growth characteristic measurement, especially because germination test might overestimate the seed potential compared to the seed responses under harsh field conditions [5]. Seed germination capacity and viability are influenced by environmental conditions during seed development, maturity levels at harvest and environment conditions during storage. The physiological and biochemical determination involves conductivity measurement, staining, and enzyme activity determination [6]. However, all of these tests are time consuming, destructive and require experienced seed analysts and special chemicals and lab arrangements.

One of the main constraints in seed production is the heterogeneity of the seed lots used during planting stage which affects growing practices and the optimum harvest time due to differential maturity of the seeds [7, 8]. Moreover, seeds undergo many forms of physiological and physicochemical alterations during storage, called ageing leading to loss in seed viability. The rate at which the seed ages depends on its ability to resist degradation and on its protective mechanisms [9]. Seed ageing is now well recognized as the major cause of reduced vigor and viability, which involves the process of deterioration and culminates in complete loss of the ability to germinate. Seed deterioration is accompanied by a cascade of physiological and biochemical perturbations resulting in reduced overall germination performance, lower speed and uniformity of germination, inferior seedling emergence and growth, reduced storability, as well as susceptibility to environmental and biological stresses, thereby resulting in a large number of abnormal seedlings and poor plant development. The low viability of seeds may also result from pest infestation or damage during drying, storage and/or any other postharvest processes. The ideal strategy for improving the overall quality in a seed lot is to screen out damaged, abnormal and non-viable seeds [6] to increase the seed uniformity and guarantee optimized plant growth protocols and yield production on farms. Segregating damaged, infected and diseased seeds from the sound ones will considerably increase the quality and the economic value of a seed batch. Thus, knowledge regarding seed vigor and viability is extremely significant for optimizing a future profitable production of cowpea.

Nowadays, the highest priority is to develop an automated and more accurate technique that minimizes human interference and be adaptable under different working conditions for quantify several seed features necessary for germination, viability and vigor testing in a wide range of crop species [4, 10, 11]. In tandem with chemometric approaches, spectral techniques have shown prospects for evaluating seed quality by predicting viability [8, 12–14], detecting damages [6], assessing chemical composition [15] and detection of pest infestation [16]. Although near infrared spectroscopy (NIRS) technique allows classification of individual seeds according to specific attributes without altering their properties, availability of spatial distribution of these attributes within a seed is completely neglected. The conventional color imaging systems can provide this absent spatial information with a number of essential quality parameters for every single seed within a seed population such as area, perimeter, length and width, shape and surface color. However, color imaging systems do not provide detailed information about the chemical composition, structure, vigor and other internal features of the seeds [17]. Hence, spectral imaging technology was emerged as a multidisciplinary task to bridge spectroscopy, imaging and machine vision technologies for simultaneous measurement of various seed traits. As a hybrid of imaging and spectroscopy techniques, spectral imaging has the ability to integrate both spectral and spatial information for visualizing structural details of specific compounds within a seed. The number of wavelengths used during image acquisition by multispectral imaging system is much fewer compared with those used with hyperspectral imaging systems. Besides its great ability in evaluating the overall quality parameters of seed lots, computer-integrated multispectral imaging system can be used also as a tool to test and evaluate individual seeds. The technique has been used successfully for the prediction of seed viability of castor beans [18], detection of seed health in spinach and wheat [19, 20], cultivar and variety discrimination in tomato, maize and rice [21–23], defect detection in maize [24], detection of mold and insect infestation in sunflower [25] and prediction of germination ability in spinach [26].

In fact, it is a very optimistic approach to find a technology that can be implemented to determine in advance which seeds are able to produce normal plants and which ones are dead or produce abnormal plants. To the best of our knowledge this is the first study to integrate computer-vision and multispectral imaging systems in combination with chemometric multivariate analysis for non-destructive quality estimation of single/individual cowpea seeds. Thus, the main aim of this study was to employ computer vision and multispectral imaging with linear discriminant modeling to differentiate between aged and non-aged seeds and the viable and non-viable seeds and explore the possibility of discriminating germination speed of individual seeds as an indication of seed vigor.

## Methods

### Description of seed samples

A total of 501 cowpea (*V. unguiculata*) seeds produced in Egypt in 2017 of a variety authorized by the Egyptian Ministry of Agriculture and Land Reclamation were used in these experiments. The seeds with initial moisture content of 12.03% (w.b.) were kept in water-proof bags in a storage room with an average temperature of 10 °C and relative humidity of 50% till the time of image acquisition in September 2018. To create various seed classes, seeds were divided into sub-samples and were liable to controlled deterioration in which seeds were artificially aged (AA) for different periods to produce enough nonviable seeds required for developing robust classification models and to produce seeds that germinate at different periods. Seeds were spread in a single layer on the surface of a bronze wire mesh placed on the top of a box (22.5 × 19 × 7 cm) containing 40 ml (1 cm deep) of deionized water. The boxes were then tightly covered with lids and then placed in an incubating chamber for 24, 48, 72 and 96 h (AA24, AA48, AA72 and AA96) at 45 °C and 98 ± 2% RH. After incubation at each ageing time, the seeds were drawn from the chamber, cooled down and air-dried for 5 h to bring them back to their original pre-aged moisture content before conducting spectral measurements. The aim of artificial accelerated ageing procedure is to decrease the activity or completely deactivate the growth hormones and enzymes such that no embryo cell division or multiplication occurs resulting in seeds with different viability and germination capacity.

### Germination test

Seeds of different ageing periods were first placed over filter paper in petri dishes (25 seeds each). After adding 40 ml of deionized water to each dish, all petri dishes were kept in an incubator at 25 °C for 3 h of imbibition. One multispectral image was acquired for imbibed seeds in each dish. After the acquisition of multispectral images, the imbibed seeds were carefully transferred from the petri dishes to the automatic germination tool supported with a computer vision system to automatically acquire color images of seeds every 1 h. As fully described by [27] and [28], the automatic computer-vision germination tool allows seed germination with continuous watering at accurately controlled temperature of 25 °C (± 0.5 °C). The accompanied computer vision devices along with its image processing routines allow recording germination parameters of individual seeds in terms of radicle protrusion and embryonic axis elongation. Over 4 days, the automated germination device analyzed all the 501 individual seeds simultaneously where the seeds with radicle protrusion longer than 2 mm were considered as germinated seeds. Afterwards, all seeds were then transferred to wet pleated filter paper in standard germination boxes (17 cm length × 11 cm width × 14 cm height). The boxes were then stored in a growth cabinet adjusted at 25 °C and 70% RH for a 16/8 (light/dark) hour photoperiod, and checked daily for 8 days of growth. In accordance to the standard method of the International Seed Testing Association (ISTA), the seedling growth status was evaluated under a standard experimental environment by the professional personnel of the National Seed Testing Station (SNES, GEVES, France). To be considered as a normal seedling, all seedling structures such as cotyledons, primary leaves, terminal bud, epicotyl, hypocotyl and roots should be clearly visible and intact. Seedlings which show damage or missing of these particular parts were considered as abnormal seedlings.

### Seed classes

According to germination data extracted from automatic computer-vision germination device as well as the growth data, the cowpea seeds were then classified into different categories based on ageing, germination status, seedling condition and speed of germination. Based on ageing process practiced initially on the seeds, they were first classified either into two groups (Non-aged & Aged) or into five subgroups (Non-aged, AA24, AA48, AA72 and AA96) corresponding to seeds aged for 24, 48, 72 and 96 h, respectively. Based on the data extracted from the automatic computer-vision germination device, seeds were classified into two groups (Germinated and Non-Germinated). Also, based on the start of germination and commencement of radicle protrusion within 48 h after sowing, seeds were classified into three classes (Early germinated, Medium Germinated & Dead). Finally, according to the seedling development after growth, seeds were classified into two classes (Normal & Abnormal). The ‘Normal’ class refers to the seeds that were able to produce normal seedlings and the ‘Abnormal’ category refers to the seeds that produced seedlings with damages, shredding or missing of shoots or roots of seedling structures (i.e. cotyledons, primary leaves, terminal bud, epicotyl, hypocotyl and roots). Although such seeds were able to germinate, they exhibited a lack of vigor and would not survive under field conditions. For this reason, the abnormal and dead seeds were lumped together as ‘Abnormal’ class [29]. In general, all seeds were split into two sets: a training set (n = 401 seeds) used for developing the discrimination models and a validation set (n = 100 seeds) for validating the developed models (Table 1).

**Table 1:** Final number of seeds (spectra) for each class utilized in the training and validation data sets

Classification type	Training set			Validation set		
	Class 1	Class 2	Class 3	Class 1	Class 2	Class 3
Ageing	81	320		19	81	
Germination	288	113		76	24	
Seedling condition	195	206		42	58	
Start of germination	90	167	94	24	42	34

### Multispectral imaging system

A multispectral image for a seed sample (25 seeds each) in each petri dish was acquired using VideometerLab3^®^ device (Videometer A/S, Hørsholm, Danemark) operated in the reflectance mode in the ultraviolet (UV), visible (VIS) and shortwave near infrared (NIR) regions with a static horizontal orientation. The device consists of a hollow integrating sphere internally painted with a white titanium coating to provide uniform scattering, high diffusing effect and minimum specular reflectance. Along the internal equator of the sphere, a series of monochromatic light-emitting diodes (LEDs) at twenty non-uniformly distributed wavelengths (375, 405, 435, 450, 470, 505, 525, 570, 590, 630, 645, 660, 700, 780, 850, 870, 890, 910, 940 and 970 nm) were mounted side by side. The LEDs with such a narrow-band spectral radiation intermittently flashed one by one and a monochromatic image of the sample was acquired at a specific wavelength by a top-mounted CCD camera, resulting in a monochrome image at each wavelength with 32-bit floating point precision for each LED type. Hence, when a sample was illuminated successively by the twenty emitting LEDs, a cube image was obtained with a spatial dimension of 2056 × 2056 pixels and 20 bands in the spectral dimension with a spatial resolution of 0.0432 mm/pixel. Generally, before acquiring multispectral images, the system was fully calibrated radiometrically and geometrically by using three successive plates: a white one for reflectance correction, a dark one for background correction and a doted one for geometric pixel position aligning calibration, followed by a light setup calibration [30].

### Image preprocessing and data extraction

**Fig. 1:**
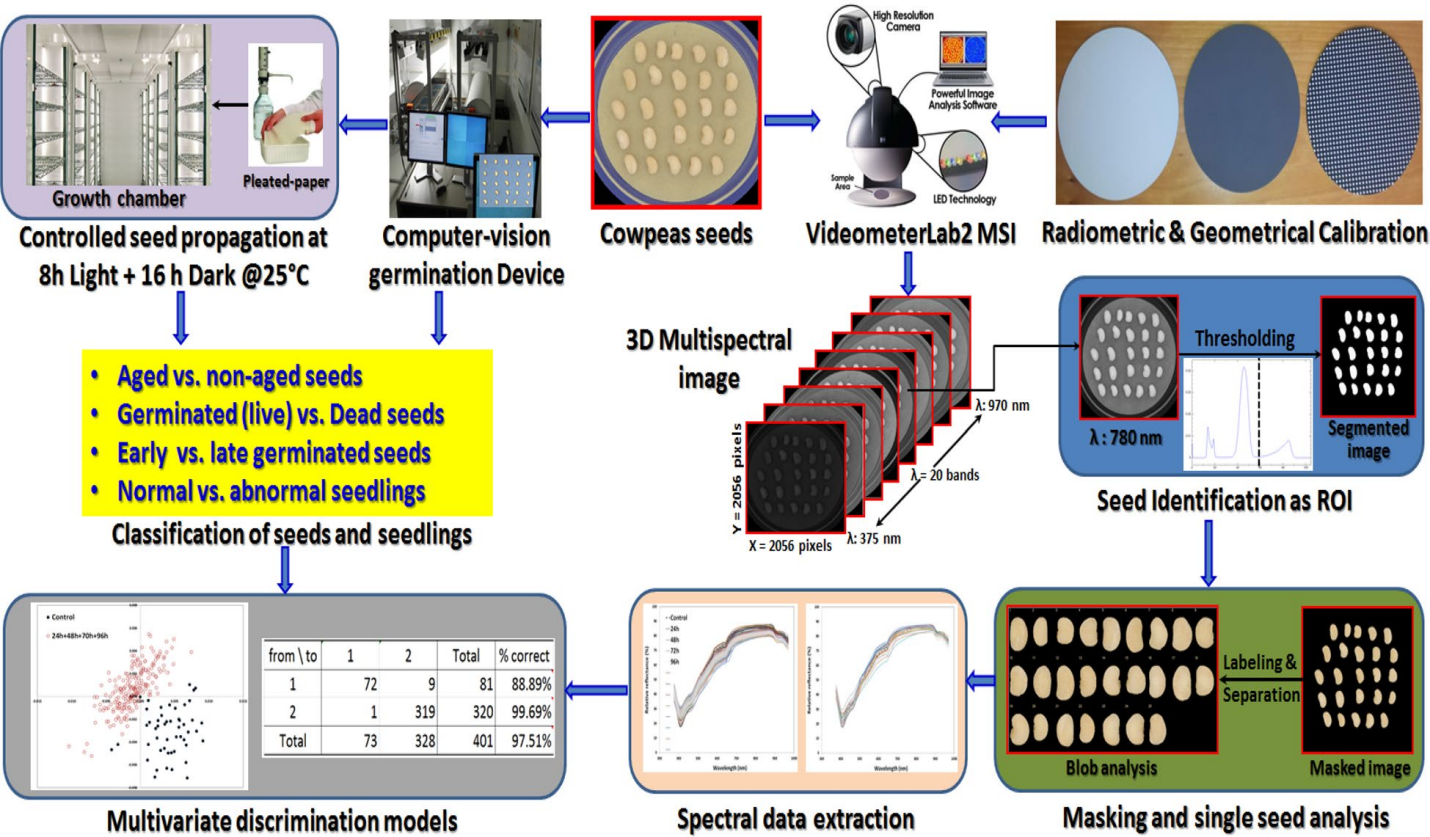
All key steps involved in processing multispectral images for extracting spectral information of the seeds, preparing germination data and building the multivariate discrimination models

The main objects appeared in the acquired multispectral image $I_{ijk}$ are the 25 cowpea seeds in addition to some other objects, such as the Petri dish and its surrounding background that should be removed from the image before going forward in extracting spectral information of the individual seeds. Thus, a preliminary image-processing step was needed to segment the acquired image $I_{ijk}$ and produce an image mask $M_{ijk}$ where only the cowpea seeds were isolated from non-seed pixels. By careful inspection of every single band in the multispectral image, it was obvious to discover that the cowpea seeds at band number 14 (780 nm) appeared as bright objects in opaque background. Hence, the image band at λ = 780 nm was segmented by a simple thresholding producing white objects with pixel values equal to 1 representing the seeds in black background with pixel values equal to 0. These white objects represent the main regions of interest (ROIs) that define the locations of the seeds achieved by thresholding the spatial dimension of band λ780 nm as described by Eq. (1):

1 $$M_{ijk} = \left\{ {\begin{array}{*{20}c} 0 & {if\;I_{ij\lambda } \le T} \\ 1 & {if\;I_{ij\lambda } \le T} \\ \end{array} } \right.$$

where *λ* corresponds to a wavelength of 780 nm, and an empirically determined threshold T was used at this wavelength band. The resulting binary image was used as a mask to identify the seed pixels in the image as the main regions of interest (ROIs). This mask was applied for all bands (from λ375 to λ970 nm) in the multispectral image highlighting only the seeds in black background in all bands. Next, all seeds were collected in a blob database from which different attributes of the seeds such as color, dimensions, texture, shape and main spectral features of all individual seeds appeared in the image could be extracted. The extracted spectral signatures of the seeds represent the mean intensity of the reflected light at each single wavelength calculated from all seed pixels in the image. Hence, the mean reflectance spectrum of any seed in the image was represented by 20 values calculated by averaging the intensity of pixels within the ROI of this seed at the 20 bands from λ375 to λ970 nm. In total, 501 average spectra representing the spectral signatures of the 501 cowpea seeds involved in this study were saved and then congregated altogether in one matrix (X) to be correlated with their corresponding germination data (Y). Figure 1 shows all key steps involved in the procedure of processing multispectral images for extracting spectral information of the seeds and building the multivariate classification models.

### Multivariate data analysis

To identify the patterns hidden in the extracted spectral data of all seeds, principal component analysis (PCA) was carried out as an explorative multivariate data analysis technique commonly used to get an overview of the systematic spectral variation in the data and to explore the possibility of grouping the seeds of similar spectral profiles. Data trending, detection of outliers and the relationship between seeds and wavelengths (variables) could be easily realized using PCA [31]. Thus, spectral data for all seeds were arranged in a matrix X where the rows represent the observations (the seeds) and the columns represent the variables (20 wavelengths). Hence, PCA was performed on this spectral data matrix and the resulting scores and loadings were observed and plotted to identify outliers and data trending.

Next, it was necessary to utilize spectral signatures of the seeds to develop multivariate discrimination models for seed classification to different categories based on their germination data. As the seeds were split into training and validation sets as shown in Table 1, the linear discriminant analysis (LDA) classification models were developed using the training (calibration) set and the models obtained were validated using the independent validation set, which was not used during building the models. To reduce the potential overfitting, the LDA models were developed under cross-validation using leave-one-out cross-validation method in which one seed was taken out at a time and the LDA model was built for the remaining seeds. The model was then used to classify the seed left out, and the same routine was repeated until all seeds removed once [32]. For developing a model using LDA method, the spectral data at all wavelengths of all seeds arranged in the X matrix (the predictors) were related with the data resulting from the germination tests saved in Y vector (the response variable). This vector contains dummy values that correspond to each seed group resulting from the germination tests. For two-class classification (e.g. Aged vs. Non-aged; Germinated vs. Non-Germinated or Normal vs. Abnormal), the categorical values in Y vector take either ‘zeros’ (for Aged, Germinated or Normal class) or ‘ones’ (for Non-Aged, Non-Germinated or Abnormal class). For three-class classification (e.g. Early, Medium & Dead), the dummy values in Y vector take either ‘zeros’ for Early germinated class, ‘ones’ for Medium germinated class or ‘twos’ for Dead seeds class. In general, the classical statistical approach of LDA computes the optimal transformation (projection), which minimizes the within-class distance and maximizes the between-class distance simultaneously, thus achieving maximum discrimination. The LDA model assesses new synthetic variables called “discriminant factors”, which are linear combinations of the all wavelengths (predictors), and allows a better separation of seed classes [33]. Then, the individual seeds can be assigned to one of the pre-defined classes. The unknown seed will be assigned to a certain class if it had a closer distance to such a class. A series of parameters can be used to evaluate the performance of classification models, such as sensitivity, specificity, and classification error [34]. In this study, the accuracy of LDA models in correctly classifying the seeds in their pre-defined classes was evaluated by using either reporting the number of misclassified seeds in each classification or by using the percentage of the overall correct classification (OCC). Thus, the recognition rates for new samples in the validation set, defined as the proportion of samples identified correctly to that of the total number in the validation set, were computed for each discriminant model using the following equation.

2 $$Accuracy = \frac{Correctly\;classidied\;seeds}{Total\;number\;of\;seeds} \times 100\%$$

Image processing and spectral data extraction were performed using the VideometerLab3 software version 1.6 (Videometer A/S, Hørsholm, Denmark) and the multivariate analyses and related statistics were performed using an in-house written script by Matlab^®^ version 7.7.0. R2008b (The Mathworks Inc., Natick, Massachusetts, USA).

## Results

### Spectral overview of aged and non-aged cowpea seeds

**Fig. 2:**
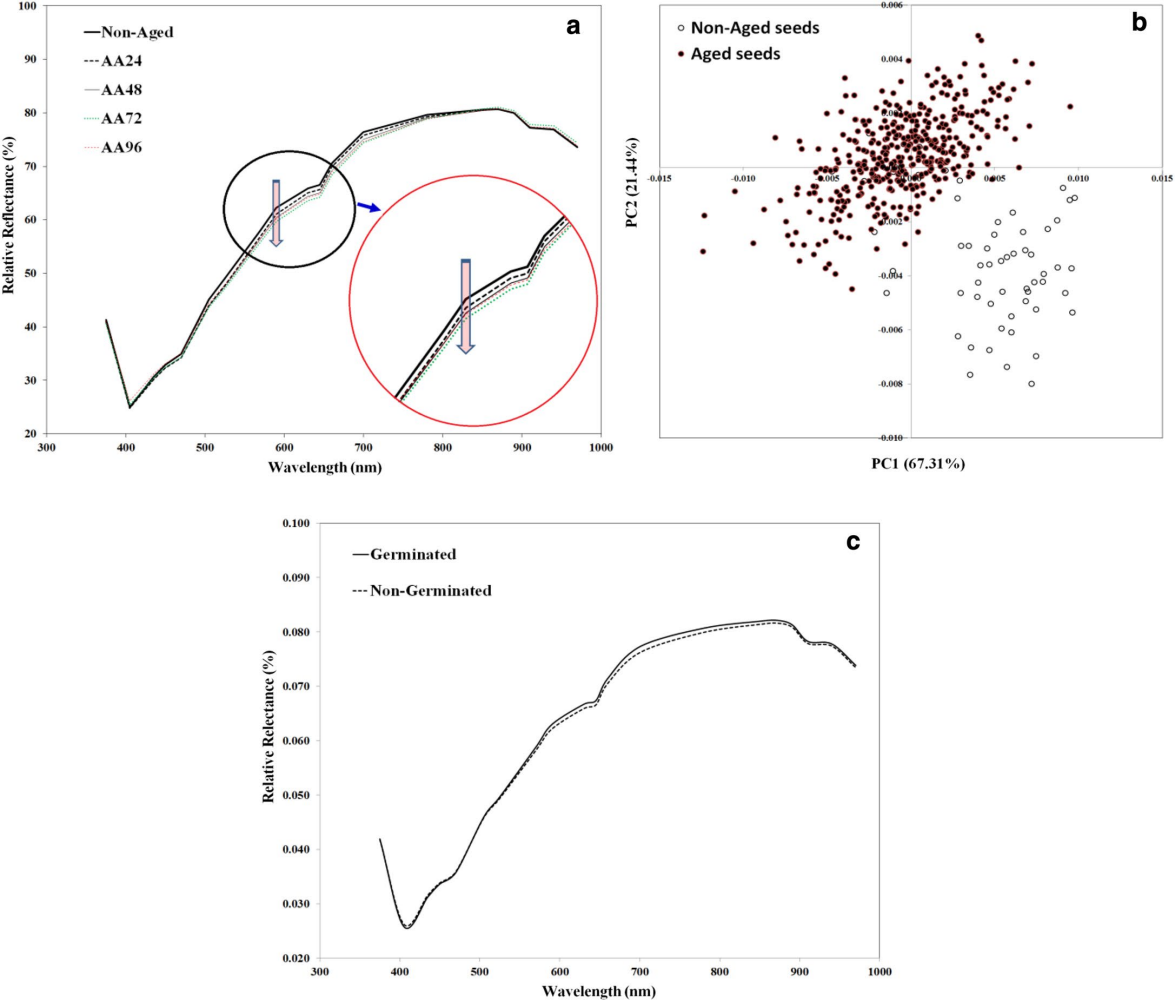
**a** Main reflectance signatures of non-aged (control) and aged seeds for different periods of artificial accelerated ageing (24, 48, 72 and 96 h), **b** PCA score plot of the raw spectral data of all cowpea seeds showing differentiation between aged and non-aged seeds, **c** main reflectance signatures of germinated and non-germinated seeds despite artificial accelerated ageing implemented

The spectrum of any pixel in the multispectral image is normally presented as a plot to show the intensity of such a pixel at different wavelengths from the UV (375 nm) to the shortwave near infrared (970 nm) range. Averaging the spectra of all pixels belonging to one seed (only one ROI or one blob) represents the spectral signature of such a seed. Any change in this spectral signature means that this seed received a peculiar change in its physicochemical attributes. The typical average spectra of non-aged (control) seeds and those received different periods of artificial accelerated ageing (AA24, AA48, AA72 and AA96) are presented in Fig. 2a. Although the aged and non-aged seeds exhibited similar spectral patterns (peaks, valleys and shoulders) in the UV, Vis and NIR regions, spectral data from non-aged seeds (the control) showed some variations in terms of reflectance intensity especially in the spectral range from 505 nm to 780 nm compared to the aged seeds (Fig. 2a). However, the differences between average spectra of aged and non-aged seeds are comparatively smaller. Despite ageing treatments, the reflectance intensity for aged and non-aged seeds in the wavelength range of 375–470 nm are almost alike, but differs when higher wavelength range were employed. It was also obvious to notice that the reflectance values of cowpea seeds decreased in the spectral range from 505 nm to 780 nm when the seeds were liable to different degrees of artificial accelerated ageing. This part of the spectrum could also contain interesting information about the seed properties. In the NIR region (i.e. from the 780 to 970 nm) the opposite was observed, and the aged seeds tended to present higher reflectance intensities compared to the non-aged seeds. The PCA performed on the spectral data of the seeds could be used to accentuate this finding. The goal of PCA was to extract the important information from the raw spectral data to represent it as a set of new orthogonal variables called principal components, and to display the pattern of similarity among seeds as points in a plot. It was noticed that the first three principle components explained 97.60% of the original variance among seed spectra with 67.31, 21.44 and 8.85% for PC1, PC2 and PC3, respectively. As shown in Fig. 2b, the score plot of PCA revealed that the aged seeds could be easily isolated from the control (Non-aged) seeds. A distinct separation among ageing treatments could not be observed on the score plot of PCA, but the non-aged (control) samples were discretely identifiable in the score plot whereas the aged seeds were grouped into one cluster. Despite the ageing treatments experienced on the seeds, the main spectral patterns of germinated and non-germinated were also plotted as shown in Fig. 2c. However, the plot shows that the difference among these two categories is not obvious and this may explains why the discrimination among them using multivariate analysis modeling could be difficult.

### Discrimination models for seed classification

As explained earlier, different linear discriminant analysis (LDA) models were developed for different classification using their spectral data at the 20 wavelengths (X-matrix) and predefined class grouping of the seeds (Y-vector).

#### Discrimination between ‘Aged’ and ‘Non-aged’ seeds

The discrimination models developed for aged and non-aged seeds were established to identify whether a batch of seeds received any kind of treatments that affects their viability and germination capacity. Two LDA models were developed in this regard: the first model was a two-class model (Aged vs. Non-aged) developed generally to discriminate aged seeds from non-aged ones; and the second model was a five-class model developed to identify the severity of ageing by discriminating seeds having different ageing periods (namely: Non-aged, AA24, AA48. AA72 and AA96). The results of predicting the class membership of seeds and the overall correct classification (OCC) of these two models are shown in Table 2 for training, cross-validation and independent validation data sets.

**Table 2:** Classification matrices of the LDA models for discrimination between non-aged (control) and aged seeds using spectral signatures (at 20 wavelengths) extracted from multispectral images of cowpea seeds

Data set	Two-class classification					Five-class classification					
		Non-aged	Aged	% Correct		Non-aged	AA24	AA48	AA72	AA96	% Correct
Training (n = 401)	Non-aged	72	9	88.89%	Non-aged	75	3	3	0	1	91.46%
	Aged	1	319	99.69%	AA24	1	77	3	0	0	95.06%
	Overall correct classification			97.51%	AA48	0	0	57	3	12	79.17
					AA72	0	1	7	71	6	83.53%
					AA96	0	0	9	6	66	81.48%
						Overall correct classification					86.28%
Cross-validation (n = 401)	Non-aged	69	12	85.19	Non-aged	71	6	3	0	2	86.59%
	Aged	1	319	99.69	AA24	1	75	4	1	0	92.59%
	Overall correct classification			96.76	AA48	0	2	53	3	14	73.61
					AA72	0	1	9	67	8	78.82%
					AA96	0	1	10	7	63	77.78%
						Overall correct classification					82.04
Validation (n = 100)	Non-aged	16	3	84.21	Non-aged	17	1	0	0	0	94.44%
	Aged	0	81	100	AA24	0	17	0	2	0	89.47%
	Overall correct classification			97.0	AA48	0	1	20	2	5	71.43
					AA72	0	0	1	15	0	93.75%
					AA96	0	0	1	0	18	94.74%
						Overall correct classification					87.00

The results revealed that the two-class LDA model was very accurate in distinguishing aged and non-aged seeds with an overall correct classification of 97.51, 96.76 and 97.0% for training, cross-validation and independent validation data sets, respectively. The result of the five-class LDA model was reasonably good but lower than that of the two-class LDA model. The percentage of the overall correct classification in training, cross-validation and validation sets was 86.28, 82.04 and 87.00%, respectively. In the independent validation set, the model was capable of differentiating seeds aged for 24, 48, 72 and 96 h with overall correct classification of 89.47%, 71.43%, 93.75% and 94.74%, respectively (Table 2).

#### Discrimination between ‘Germinated’ and ‘Non-Germinated’ seeds

As a complementary work of discriminating aged seeds from the non-aged seeds, it was very important to differentiate between the germinated seeds (i.e. viable seeds) and the non-germinated seeds that could be decayed, dead of delayed in germination under the effect of ageing. The time series images acquired by the automated computer-vision germination device showed clearly which seeds were germinated (live) and which ones failed to germinate during the first 4 days after sowing. Hence, a new linear discriminant analysis model was built to discriminate between germinated and non-germinated seeds in training data set under cross validation (n = 401) and the resulting model was used to classify the seeds in an independent validation set (n = 100). As shown in Table 3, this LDA model correctly classified the germinated seeds with an accuracy of 92.71, 89.93 and 93.42% in training, cross-validation and validation sets, respectively. Meanwhile, the performance of the model in detecting non-germinated seeds was not good enough with an accuracy of 53.98, 51.33 and 41.67% in training, cross-validation and validation sets, respectively. However, the overall accuracy of the model in classifying the seeds based on germination criterion was reasonably good with an overall correct classification of 81.80, 79.05 and 81.0% in training, cross-validation and validation sets, respectively (Table 3). As the predictions of germinated and non-germinated seeds in the validation set was very much similar to the training set, the model could be used safely in this task for discriminating viable from dead seeds. Generally, in all classification modeling outlined in this work, a posterior membership probability threshold of 0.5 was used to recognize the seeds’ predicted classes. A seed was assigned to a certain class if its membership probability was greater than 0.5. In case of aged/non-aged classification, the seeds had average membership probability of 0.996 ± 0.024 for being aged class and 0.946 ± 0.107 for being non-aged class. Similarly, in case of germinated/non-germinated classification, the seeds had a membership probability of 0.858 ± 0.123 for being germinated class and 0.737 ± 0.139 for being non-germinated class. Despite the membership probability calculations, the squared distances between the spectrum of a seed and the centroid of each class were calculated. A seed was assigned to a certain class when its spectrum had the minimum squared distance to this class.

**Table 3:** Confusion matrices of the LDA model for class membership of ‘Germinated’ and ‘Non-Germinated’ cowpea seeds in training, cross validation and validation sets

Group	Training set (n = 401)			Cross-validation set (n = 401)			Validation set (n = 100)		
	Germinated	Non-Germinated	% Correct	Germinated	Non-Germinated	% Correct	Germinated	Non-Germinated	% Correct
Germinated	267	21	92.71	259	29	89.93	71	5	93.42
Non-Germinated	52	61	53.98	55	58	51.33	14	10	41.67
Overall correct classification			81.80			79.05			81.00

#### Discrimination between ‘Normal’ and ‘Abnormal’ seedlings

Table 4 shows the performance of another LDA model built for classifying cowpea seeds into two categories: (1) seeds that are able to produce normal seedlings and (2) seeds that produced abnormal seedlings due to deformed essential structures or missing one or more of their essential structures. The results revealed that the accuracy of the model was moderate with overall classification accuracy of 68.08, 64.34 and 62.00% in training, cross-validation and validation datasets, respectively. To improve the performance of such a model, more seeds should be involved to include all possible variation in considerations.

**Table 4:** Confusion matrices of the LDA model built for class membership of cowpea seeds to produce ‘Normal’ and ‘Abnormal’ seedlings in training, cross validation and validation sets

Group	Training set			Cross-validation set			Validation set		
	Normal	Abnormal	% Correct	Normal	Abnormal	% Correct	Normal	Abnormal	% Correct
Normal	130	65	66.67	124	71	63.59	26	16	61.90
Abnormal	63	143	69.42	72	134	65.05	22	36	62.07
Overall correct classification			68.08			64.34			62.00

#### Discrimination of cowpea seeds based on starting of germination

Because the automated computer-vision germination device monitors the seed conditions every 1 h, seeds were categorized into three groups: seeds that germinated within 48 h (vigorous or ‘Early’ germinated seeds), seeds that germinated within 72 h (‘Medium’ seeds) and ‘Dead’ seeds (unviable or decayed). This categorization of the seeds was saved in a dummy variable (Y vector) and was linked with the original spectral signatures (X matrix) of theses seeds using another LDA model to unambiguously determine the identity of every individual seed. The LDA model was built to classify seeds to these predefined classes based on their spectral data. Comparison of these assigned classes to the original predefined classes is an indicator of the discrimination accuracy. The results of the LDA model are shown in Table 5. It was clear to notice that the model can discriminate these classes with overall classification accuracy of 77.21, 74.93 and 68.00% in training, cross-validation and independent data sets, respectively. It seems that the model significantly underperformed in the independent validation set (68.00%), although they exhibited reasonable performance during training. The accuracy of the model in identifying the ‘Early’ germinated seeds was 80.00, 77.78 and 91.67% in training, cross-valuation and validation datasets, respectively.

**Table 5:** Performance of the LDA model for the classification of all sets of seeds based on seed vigor expressed by the period to commence germination

Dataset	Early	Medium	Dead	% Correct
Training (n = 401)				
Early	72	16	2	80.00
Medium	2	137	28	82.04
Dead	2	30	62	65.96
	Overall correct classification (%)			77.21
Cross-validation (n = 401)				
Early	70	18	2	77.78
Medium	3	135	29	80.84
Dead	2	134	58	61.70
	Overall correct classification (%)			74.93
Validation (n = 100)				
Early	22	2	0	91.67
Medium	2	29	11	69.05
Dead	1	16	17	50.00
	Overall correct classification (%)			68.00

**Fig. 3:**
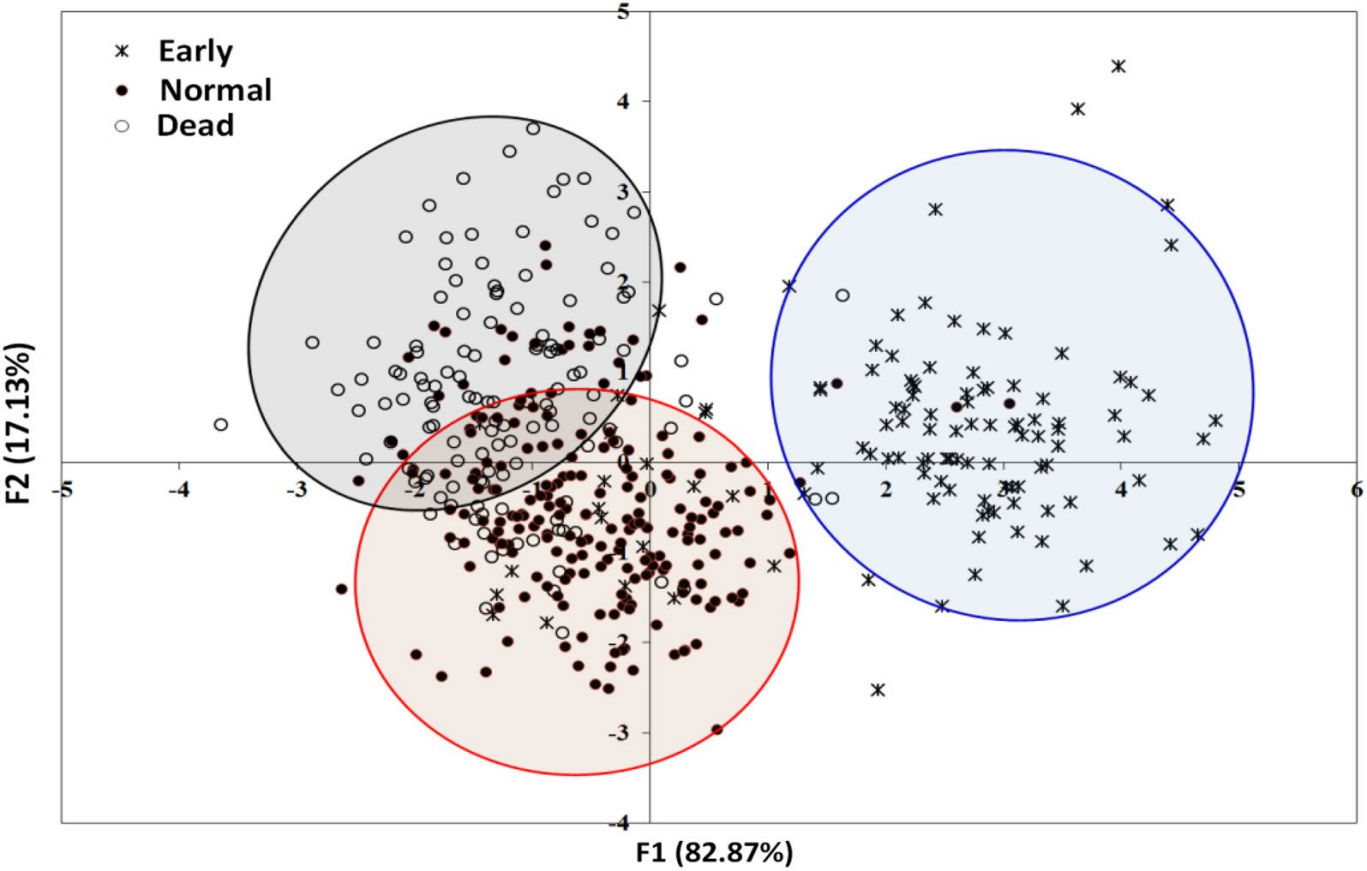
Score plot of the LDA model for discrimination cowpea seeds based the starting of germination illustrating the separation of ‘Early’ germinated seeds from the ‘Medium’ and ‘Dead’ seeds. Circles around data points were used to improve the clarity of discrimination and do not have any mathematical significance

As shown in Fig. 3, plotting the first and second discriminant factors (F1 and F2) that explained 82.87% and 17.13% of the total variance by this LDA model showed a clear discrimination among seeds of different vigor classes that started to germinate at different times. The plot showed that seeds of the three vigor classes were mostly separated and well differentiated from each other especially the ‘Early’ seeds (germinated within 48 h), which entirely located on the positive scores of F1. The ‘Medium’ and ‘Dead’ seeds could be noticeably located on the negative scores of F1. The majority of the ‘Medium’ seeds were located on the negative scores of F2; meanwhile most of the ‘Dead’ seeds lied on the positive scores of F2. However, there were some interactions between these two later classes due to partial variation among individual seeds in terms of their original physicochemical properties. In general, classification results of training, cross–validation the validation sample sets using all LDA models developed in this study are summarized in Table 6.

**Table 6:** Overall correct classification LDA models developed for different classification scenarios of cowpea seeds

No. of classes	Seed grouping	Overall correct classification (%)		
		Training (n = 401)	Cross-validation (n = 401)	Validation set (n = 100)
2	Non-aged and Aged	97.51	96.76	97.00
5	Non-aged, AA24, AA48, AA72 and AA96	86.28	82.04	87.00
2	Germinated and Non-Germinated	81.80	79.05	81.00
2	Normal and Abnormal	68.08	64.34	62.00
3	Early, Medium and Dead	77.21	74.93	68.00

## Discussion

The variations in the spectra of aged seeds are ascribed to the changes in the physical and chemical properties of such seeds. Compared with the non-aged seeds, the aged seeds presented lower reflectance (higher in absorbance) in the visible region and higher reflectance (lower in absorbance) in the NIR region. Such differences in reflectance magnitudes indicate differences in color and chemical composition between aged and non-aged seeds. This finding indicates that protein, starch, and other components in damaged seeds have changed, thus resulting in changes in the spectra [6]. The variations in the visible range (375–780 nm) can be attributed to the change occurred in the color of the seed, whereas the variations in the NIR region (780–970 nm) were due to physicochemical changes [35]. However, the discoloration that may occur in seeds due to ageing is sometime very difficult to discern with the naked eyes and it was necessary to develop a model to visualize this change in a simple form. The chemical changes occurred in cell membrane during seed ageing were responsible for a change in seed viability and vigor and generate changes in reflectance profile at specific spectral regions. It is believed that ageing is caused by a significant decrease in embryo cell division and expansion either because of unsuitable growth environment and/or cell physiology. Also, the seed food stores (starch, proteins and lipids) that all are necessary for embryo development are also affected by ageing. Deterioration of seed membranes are accompanied by changes in biochemical compounds such as increases of ethanol and fatty acids and dramatic changes in lipids, sugar, protein and starch that could be tracked all in the visible and NIR spectra. The most influencing factor in seed ageing and deterioration is the water binding within seed cells seems [36], followed by processes of protein and lipid modification [37]. Owing to the overlapping nature of the spectra and the complex chemical composition of seeds, interpretation of absorption bands is not straightforward. Therefore, the spectral assignment of the absorption bands and their interpretation is fairly complicated due to the presence of various overlapped peaks and complex chemical composition of the seeds [38]. While absorption bands at 425 nm could be assigned to the change in melanin content, the absorption bands at 940 and 970 nm could be associated with lipid and moisture contents, respectively [39, 40].

The discrimination results between aged and non-aged seeds presented in Table 2 are in agreement with those obtained from PCA illustrated in Fig. 2b indicating that the physicochemical changes occurred in cowpea seeds during ageing were reflected in the spectral signatures of the seeds that facilitate their discrimination. In fact, during accelerated ageing lipid peroxidation leads to deterioration of cell membranes and reducing seed viability. These biochemical changes may be the reason for a clear grouping between aged and non-aged seeds when performing principle component analysis and linear discriminant analysis [13]. The degree of overlapping observed between aged seeds (AA24, AA48, AA72 and AA96) could be ascribed to the differential rates of deterioration of individual seeds, which in turn could be associated to initial health, vigor and size of these seeds. The variability in seed size within a seed lot was the main source of the variation in seed deterioration during accelerated ageing because the smaller seeds deteriorate faster than larger seeds [41]. In general, the results demonstrated that the visible and NIR spectral data extracted from multispectral images of cowpea seeds contained the required chemical information for rapid discrimination of non-aged seeds and sorting out the aged seeds. This result is of great significance in improving seed quality by excluding naturally aged seeds due to severe and unstable storage conditions and those seeds severely desiccated just before harvesting. Also, the importance of this discriminant model is also very significant in predicting the optimum conditions required for preserving seed quality during storage and for making a decision on the longest duration of seed storage without deterioration.

The loss of seed quality and viability depends basically on the duration of the ageing process, conditions of storage and characteristics of species itself. These factors affect the degree of deterioration in cellular membranes and damages occurred in seed structures. It is believed that biochemical processes of lipid peroxidation are the major cause of seed deterioration during ageing and cause a decrease in seed germination and loss of viability. In addition to these damages, degradative changes in insoluble carbohydrates and protein macromolecules led to a reduction in seed capacity to absorb water, imbibition ability and germination capacity. The speed at which the seed ageing process takes place depends on the seed’s ability to resist such deteriorative changes, which are species-specific [42]. Based on the cultivar, longer ageing period is expected to cause more severe changes in seed integrity and affect the seed overall quality (Table 3). By the way, the task of any proposed method of seed quality evaluation is to judge the seed integrity based on different morphological and physicochemical parameters. This finding is in agreement with those obtained in different seed species. In this regard, multispectral imaging in the visible and near infrared regions (375–970 nm) with 19 wavelength bands has been tested in viability detection of castor (*Ricinus cummunis* L.) seeds. The results revealed that viable seeds were distinguished from dead seeds with accuracy of 92 and 96% in the calibration and validation data sets, respectively. This result was confirmed by tetrazolium test performed on cut seeds, in which a similar pattern as for the whole seed was observed [18]. Moreover, the main challenge is to utilize the spatial and spectral information extracted from multispectral images of cowpea seeds in predicting the ability of such seeds to produce normal seedlings with healthy seedling essential structures (roots, cotyledons, primary leaves, terminal bud, epicotyl and hypocotyl). An abnormal seedling is defined when one or more of the essential seedling structures is impaired or missed. Such abnormal seedlings are incapable of giving normal growth and are therefore incapable of developing into healthy plants in the field. In this regard, the results revealed that the accuracy of the LDA model designated to discriminate between seeds that produce normal seedling and seeds that developed abnormal seedling (Table 4) was moderate with overall classification accuracy of 68.08, 64.34 and 62.00% in training, cross-validation and validation datasets, respectively. In fact, this model needs some improvements by utilizing some other models such as quadratic discriminant model or non-linear models could be examined to enhance the overall classification accuracy. Also, extracting some textural features of the seeds at certain wavebands could have positive effect in increasing the accuracy of the classification models. In this regard, Shetty et al. [26] investigated the potential of multispectral imaging in predicting germination capacity and germ length of spinach seeds. They reported that combining spectral data such as the mean, median, minimum, maximum and standard deviation values of reflectance intensities of the seeds with texture features such as entropy angular second moment, contrast and correlation improve the accuracy of the classification models. Their results revealed also that larger seeds had not only higher germination potentials but also bigger germ length compared with smaller seeds.

**Fig. 4:**
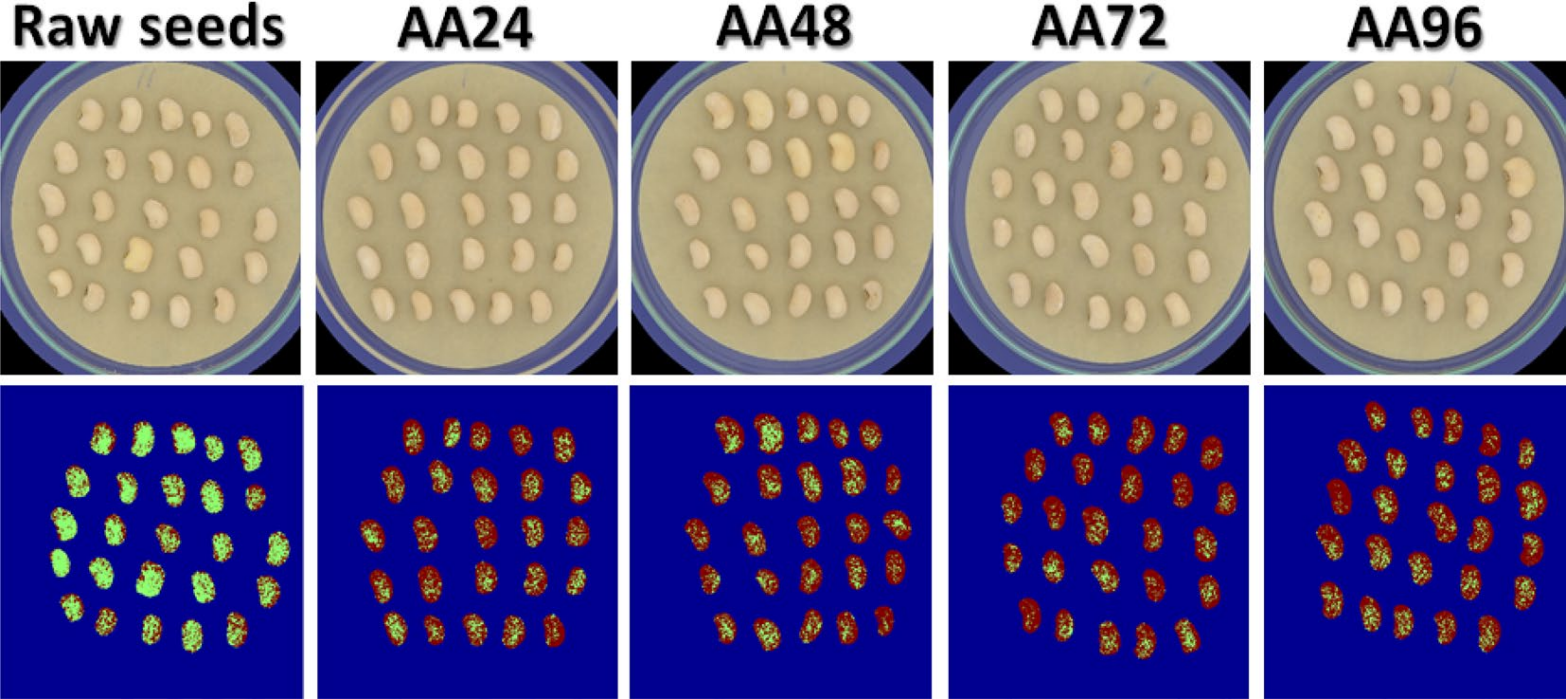
Application of LDA model for discrimination between non-aged seeds and those seeds aged for 24, 48, 72 and 96 h. The first raw shows the original color images of the seeds and the second raw visualizes the classification results after applying the LDA model in every single pixel in the images. Green color denotes the ‘Non-aged’ class and red color denotes the ‘Aged’ class

The accelerated ageing process results in seeds characterized by reduced ‘speed’ of germination, and poor seedling development. The term vigor is used to describe the physiological characteristics of seeds that control its ability to germinate ‘rapidly’ in the soil and to tolerate unfavorable environmental conditions. Apparently, the initial stage of seed growth is very significant for further development and survival under severe environmental conditions. As the extreme conditions of artificial seed ageing caused significant decline in germination, the high vigor seeds are expected to tolerate high temperature and humidity (i.e. ageing conditions) while retaining their capability to produce normal seedlings [42]. The seeds high in vigor generally provide early and uniform stands, indicating that the seeds have the potential to produce vigorous seedlings under favorable conditions. Therefore, in this study, the time taken by a seed to start germination could be considered as a parameter for seed vigor, and seeds that showed early germination were considered to be more vigorous [9]. In this regard, the computer vision system was extremely significant in identifying the seeds which germinated first. The computer-vision system imaged the seeds repeatedly in the same position allowing algorithms to identify minor changes, and to disregard changes associated with mould or seed expansion due to water uptake, which should not be scored as germination [10]. In fact, identifying the most vigorous seeds that early germinated within 48 h after sowing is very important from different prospective because quick formation of assimilation surface and root system in early stage of plant life gives these plants certain advantage during later stages of development and growth. As some of the aged seeds germinate at later stage, this indicates that the seeds usually loss their vigor as a result of seed degradation, followed by loss of germination and viability. As the ageing process of the seeds affects their viability, germinating capacity and vigor, it was necessary to visually discriminate the aged and non-aged seeds in an easy way. However, visual inspection of cowpea seeds indicated that it was very difficult to discriminate between the aged and non-aged seeds based only on their color as shown in the first row of Fig. 4. Also, the absorption peaks of the visible NIR spectra of the seeds were very broad and overlapping (Fig. 2a), making discrimination impossible based on single wavelength visualization due to large hidden information in all spectral range. To exploit the full power of multispectral imaging, it was extremely important to recognize whether a cowpea seed was aged or not by transferring the discriminant model to every single pixel in the image to visualize the difference of seed categories in the images. The LDA model only keeps all important wavelengths necessary to distinguish the categories of cowpea seeds receiving different ageing regimes and discards the other collinear wavelengths that are unnecessary in discrimination. The three-dimensional multispectral image cube $I_{ijk}$ acquired at 20 spectral bands was unfolded first to a two-dimensional matrix $M_{{\left( {i \times j} \right)k}}$ in which each row represents the spectral signature of one pixel in the original image. The linear discriminant function resulting from the LDA model was then applied to this two-dimensional matrix $M_{{\left( {i \times j} \right)k}}$ to produce a one-row vector $R_{{\left( {i \times j} \right)}}$ having distinct values corresponding to the predicted class for every row. A simple color mapping was then applied on the resulting values of this vector $R_{{\left( {i \times j} \right)}}$ in which the blue color represents ‘Background’, green represents ‘Non-aged’ class and red represents the ‘Aged’ class. The mapped vector was then refolded back to a 2-D form for visualizing different seed classes appeared in the image. The resulting images were called the ‘*Classification images*’, which were very easy to understand and visually simple to interpret as shown in the second row of Fig. 4. The advantage of these developed LDA models is that they are not exclusively dependent on the VideometerLab3 multispectral imaging system used in this study as the other built-in transformation tools reported by other authors [18, 20, 22, 23], but it can be freely used in any multispectral imaging systems utilizing the same configuration of the image acquisition scenario. Although the sample size was different in each class, this is not necessarily to negatively affect the accuracy of the LDA models in every individual class as there is no reliable empirical evidence to support the claim that unbalanced data set has a negative effect on the performance of LDA [43]. For instance, in case of the LDA model developed for the discrimination of cowpea seeds based on the starting of germination to three classes (Early, Medium and Dead), one can find that the number of samples in these classes were unbalanced with 90, 167 and 94 seeds in the training set and 24, 42 and 34 seeds in the validation set (with the ‘Medium’ class having a large number of samples compared to the other two classes). However, the performance of the LDA model was 80, 82, 65% in the calibration set and 91, 69.05 and 50% in the validation set. Therefore, the criterion used in this study to evaluate the accuracy of the LDA models was the overall accuracy of the model in identifying the identity of the seeds in all classes.

In fact, showing the classification maps in this representation is very important to easily detect seeds having peculiar features and eliminate them from the whole seed lots. This could be used for high throughput imaging, particularly where the identification of abnormal seeds is of great importance. Considering the short time of image acquisition and limited sample preparation, this stat-of-the art multispectral imaging method and chemometric analysis in classifying seeds could be a valuable tool for on-line classification protocols in cost-effective real-time sorting and grading processes as it provides not only morphological and physical features but also overall information for the seeds being examined.
